# Switching Multiple Sclerosis Patients with Breakthrough Disease to Second-Line Therapy

**DOI:** 10.1371/journal.pone.0016664

**Published:** 2011-02-03

**Authors:** Tamara Castillo-Trivino, Ellen M. Mowry, Alberto Gajofatto, Dorothee Chabas, Elizabeth Crabtree-Hartman, Bruce A. Cree, Douglas S. Goodin, Ari J. Green, Darin T. Okuda, Daniel Pelletier, Scott S. Zamvil, Eric Vittinghoff, Emmanuelle Waubant

**Affiliations:** 1 Multiple Sclerosis Center, Department of Neurology, University of California San Francisco, San Francisco, California, United States of America; 2 Multiple Sclerosis Unit, Department of Neurology, Hospital Donostia, San Sebastián, Spain; 3 Department of Neurological and Vision Sciences, University of Verona, Verona, Italy; 4 Department of Epidemiology and Biostatistics, University of California San Francisco, San Francisco, California, United States of America; City of Hope National Medical Center and Beckman Research Institute, United States of America

## Abstract

**Background:**

Multiple sclerosis (MS) patients with breakthrough disease on immunomodulatory drugs are frequently offered to switch to natalizumab or immunosuppressants. The effect of natalizumab monotherapy in patients with breakthrough disease is unknown.

**Methods:**

This is an open-label retrospective cohort study of 993 patients seen at least four times at the University of California San Francisco MS Center, 95 had breakthrough disease on first-line therapy (60 patients switched to natalizumab, 22 to immunosuppressants and 13 declined the switch [non-switchers]). We used Poisson regression adjusted for potential confounders to compare the relapse rate within and across groups before and after the switch.

**Results:**

In the within-group analyses, the relapse rate decreased by 70% (95% CI 50,82%; p<0.001) in switchers to natalizumab and by 77% (95% CI 59,87%; p<0.001) in switchers to immunosuppressants; relapse rate in non-switchers did not decrease (6%, p = 0.87). Relative to the reduction among non-switchers, the relapse rate was reduced by 68% among natalizumab switchers (95% CI 19,87%; p = 0.017) and by 76% among the immunosuppressant switchers (95% CI 36,91%; p = 0.004).

**Conclusions:**

Switching to natalizumab or immunosuppressants in patients with breakthrough disease is effective in reducing clinical activity of relapsing MS. The magnitude of the effect and the risk-benefit ratio should be evaluated in randomized clinical trials and prospective cohort studies.

## Introduction

It has become common in daily practice for patients with relapsing forms of multiple sclerosis (MS) who have breakthrough disease on first-line disease-modifying therapy (DMT) (interferon-beta and glatiramer acetate) to switch to other agents. Before natalizumab was available, broad-spectrum immunosuppressants such as cyclophosphamide, mitoxantrone, methotrexate and mycophenolate mofetil (hereafter grouped together and referred to as “immunosuppressants”) were often used as second-line therapies. With the re-introduction of natalizumab to the market in 2006 for patients with MS [1], [2], options for treating breakthrough disease have expanded to include switching to another first-line DMT, to an immunosuppressant, or to natalizumab. While previous studies have provided a rationale for switching to another therapy in breakthrough disease, none has evaluated the efficacy of switching to second-line monotherapy versus controls in this context [3], [4], [5], [6].

Our aim was to compare the clinical effects of three treatment strategies in patients with breakthrough disease: i) switching to natalizumab, ii) switching to an immunosuppressant, or iii) remaining on or discontinuing first–line treatment.

## Materials and Methods

### Patients' characteristics

A systematic chart review of 993 patients seen at least four times at the University of California San Francisco (UCSF) MS Center from November 2005 to November 2008 was performed by a single neurologist (TCT) to identify patients with relapsing-remitting (RR) or relapsing secondary progressive (SP) MS [7] whose physicians recommended a switch to second-line therapy due to breakthrough disease despite at least six months on first-line DMT [8]. Among these patients, we identified three groups: i) patients who switched to natalizumab (natalizumab group), ii) patients who switched to an immunosuppressant (immunosuppressant group) and iii) patients who were offered natalizumab but did not start the treatment, typically due to insurance constraints or fear of adverse effects (non-switchers). We also queried the UCSF MS Center database from 2002 (date of database availability) onward, to identify patients who switched to immunosuppressants. In all groups, the definition of breakthrough disease and the decision to recommend a switch was made by each patient's UCSF MS Center neurologist.

The date of the switch was defined as the date when the second-line therapy was started (for the natalizumab or immunosuppressant switchers) or the date when the switch was proposed (for the non-switchers). Annualized relapse rates were calculated for all patients in the last year on DMT and during the entire follow-up period.

For all patients, at least three months of follow-up after the proposed switch were required. Patients who switched to second-line therapy due to poor tolerability of first-line DMT were excluded from the study. Patients for whom meaningful confounders (such as EDSS at the time of the switch) were missing were excluded.

Relapse was defined as the development of new or recurrent neurological symptoms not associated with fever or infection lasting for at least 24 hours and accompanied by new neurological signs, following a period of symptomatic stability of 30 days [7]. Pseudoexacerbations were excluded.

This study was approved by the UCSF Committee on Human Research (CHR). As the data were extracted from a clinical database used for all patients seen at the MS clinic, authors weren't required to ask for written consent as they had a waiver of consent from UCSF CHR.

### Statistical Analyses

Characteristics of the three groups at the time of switch were compared using ANOVA, Kruskal-Wallis, chi-square or Fisher's exact tests, as appropriate. A Poisson regression model was used to compare relapse rates. Differences in the length of follow-up were accommodated using so-called offsets, while within-person correlation of relapse rates in the periods before and after switching were accounted for using a normally-distributed random intercept. The effect of switching to natalizumab or immunosuppressants was captured by interactions of the post-switch treatment with the period (post- vs pre-switch). We identified age, gender, race, EDSS at the time of the switch (baseline EDSS), disease duration, number of first-line DMTs received and time on first-line DMT before the switch as potential confounders of the effects of the treatment received after failure of first-line DMT. Accordingly, we adjusted both for the main effects of these variables, which capture their *effects on the pre-switch relapse rate*, as well as their interactions with the period, which capture their *effects on reductions in the relapse rate after switching*. We modeled non-linearities in the effects of disease duration and time on first-line DMT using restricted cubic splines. The regression analysis excluded four patients with missing baseline EDSS scores. Because the sample size was limited, in particular for non-switchers, we removed covariates from the full model with p-values >0.25. The main statistical analyses were performed using the xtpoisson command in STATA Version 10.0 (StataCorp, College Station, TX).

## Results

### Patients' characteristics

**Figure 1:**
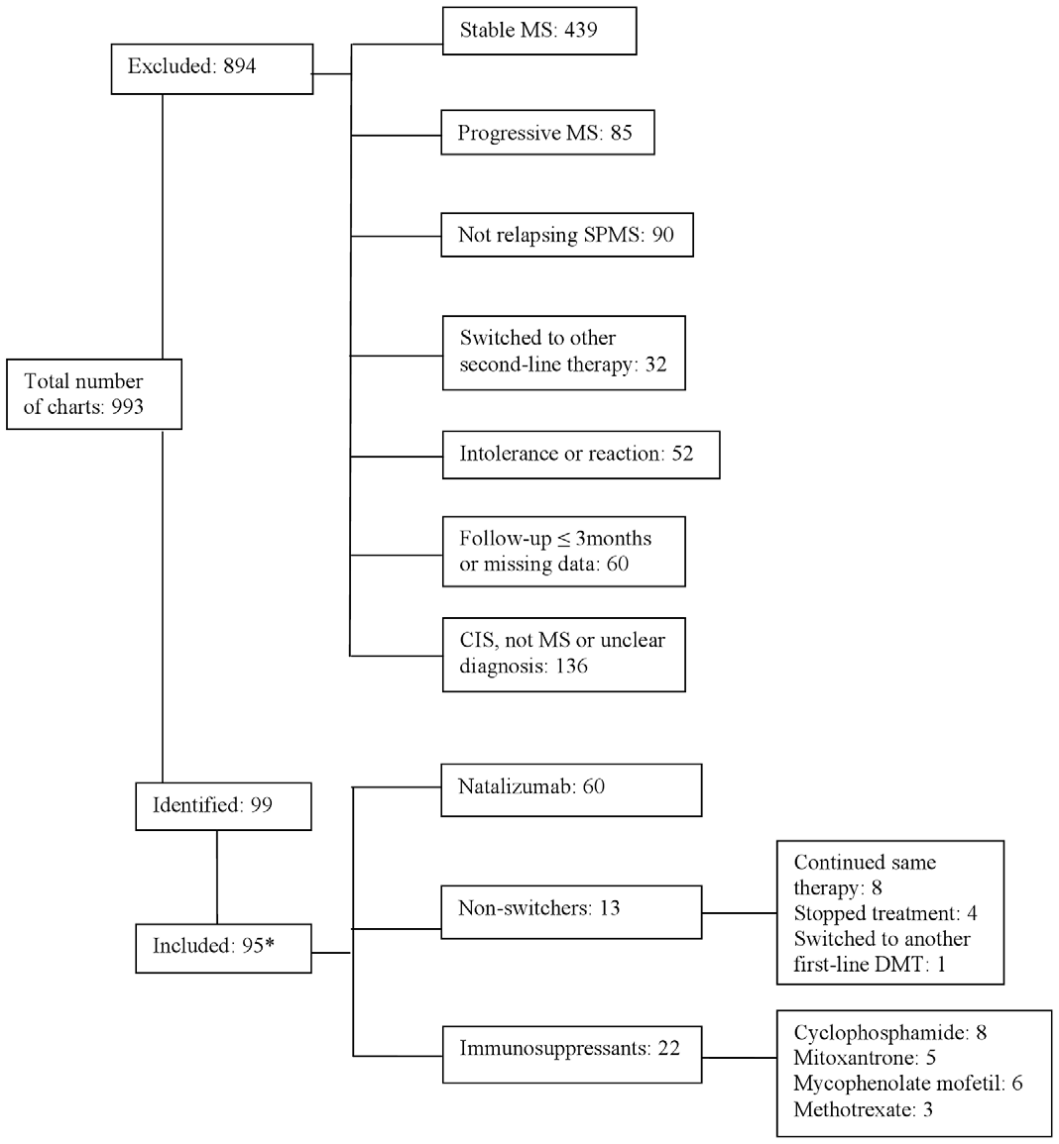
Diagram of charts reviewed. Diagram showing the total number of charts reviewed, the reason for exclusion and all included patients in the study. For those patients who switched to immunosuppressants, a detail of the immunosuppressant agents used is provided and for the non-switchers further detail is presented. *From the 99 identified patients, 4 were excluded from the statistical analyses due to missing information for important baseline characteristics.

The details regarding the inclusion or exclusion of reviewed charts are provided in Figure 1. We identified 99 patients; for the 95 who had complete data, characteristics at the time of switch are provided in Table 1. Within this group, 60 switched to natalizumab, 22 switched to immunosuppressants, and 13 were non-switchers (eight remained on the same DMT, one switched to another first-line DMT and four stopped treatment). The number of and time on first-line DMT before the switch, EDSS and percentage of those with relapsing SPMS at the time of switch, and years of follow-up after the switch differed between the groups at baseline. These results were not meaningfully changed when the four patients with missing EDSS measures were included.

**Table 1:** Patients' characteristics.

	Non-switchers n = 13	NatalizumabSwitchers n = 60	ImmunosuppressantsSwitchersn = 22	p-value
Age†	37.2 (10.8)	40.5 (10)	41.7 (10.4)	0.44
Female, % (No)	53.8 (7)	66.7 (40)	63.6 (14)	0.68
Caucasian, % (No)	69.2 (9)	73.3 (44)	77.3 (17)	0.84
Relapsing SPMS at the switch, % (No)	15.4 (2)	20 (12)	52.4 (11)	0.014
Disease duration†, years	8.9 (7.9)	11 (6.5)	11.5 (6.4)	0.5
Number of DMTs before the switch§	2 (1–3)	2 (1–3)	1 (1–3)	0.07
Years on first-line DMT before the switch†	3.4 (1.9)	5.3 (3.3)	4.1 (2.8)	0.08
Years of follow-up after the switch†	1.1 (0.6)	1.1 (0.6)	3.3 (2.4)	<0.001
EDSS at the time of switch§	2.5 (0–7.0)	3.0 (1.0–7.0)	4.0 (2.5–7.0)	0.01
ARR in the last year on DMT(range)	1.30 (0.9)(0–3)	1.32 (0.9)(0–5)	1.00 (0.9)(0–3)	0.4
Crude ARR in the whole DMT period(range)	1.14 (0.6)(0.37–2.34)	1.04 (0.7)(0–3.23)	0.95 (0.6)(0–2.4)	0.7
Crude ARR after the switch(range)	0.61 (0.9)(0–2.72)	0.38 (0.8)(0–4.01)	0.62 (0.9)(0–3.44)	0.4

† Mean (±SD).

§ Median (range).

The annualized relapse rate (ARR) during the last year of treatment with first-line disease modifying therapy, as well as the ARR for the whole period on DMT and ARR after the switch. The time of follow-up after the switch is also presented for each patient group. SPMS: secondary progressive multiple sclerosis. DMT: disease modifying therapy. EDSS: expanded disability status scale.

In the study 60 patients received more than one first-line DMT before the switch and 35 received only one: 63 patients received Avonex, 28 patients received Betaseron, 40 received Rebif and 43 received Copaxone.

### Relapse rate reductions

**Figure 2:**
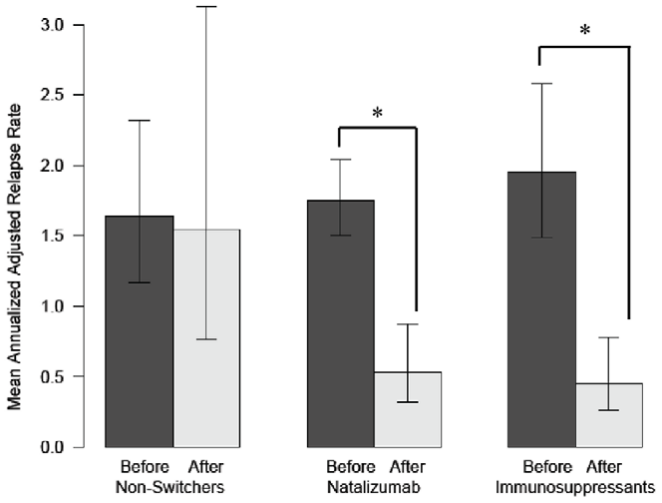
Mean annualized relapse rate before and after the switch for each group. Bar plot showing the effect of switching therapy on the relapse rate within group. The model is adjusted for baseline EDSS, disease duration and time on first-line disease modifying therapy. Bars indicate 95% CI for the mean annualized relapse rate. Non-switchers: n = 13, Natalizumab: n = 60, Immunosuppressants, n = 22. * Statistically significant.

In the pre-switch period, relapse rates were similar across the three groups (p = 0.71), after adjustment for covariates, including during the last year (data not shown). In the post-switch period, the relapse rate was reduced both among natalizumab (70%, 95%CI 50, 82%, p<0.001) and immunosuppressant switchers (77%, 95% CI 59, 87%, p<0.001) but was not substantially reduced among the non-switchers (6%, 95% CI -99, 56%, p = 0.87) (Table 2). After adjustment for the effects of covariates on reductions in the relapse rate, switching had an overall positive effect on clinical activity (p = 0.01 for heterogeneity). In pair-wise comparisons with non-switchers, the net reduction in the relapse rate was 68% among natalizumab switchers (95% CI 19, 87%, p = 0.017) and 76% among immunosuppressant switchers (95% CI 36, 91%, p = 0.004). Finally, those who were switched to immunosuppressants had a 24% reduction in the relapse rate when compared to those who switched to natalizumab (p = 0.49). Figure 2 represents the effect of the switch on the relapse rate for each group.

**Table 2:** Effect of switching on the relapse rate measured as incidence rate ratio (IRR), using a Poisson regression model, after adjusting for baseline EDSS, disease duration and time on first-line disease modifying therapy.

	IRR	95% CI	p-value
**WITHIN GROUP COMPARISON**			
Non-switchers (n = 13)	0.94	(0.44,1.99)	0.87
Natalizumab switchers (n = 60)	0.3	(0.18,0.50)	<0.001
Immunosuppressant switchers (n = 22)	0.23	(0.13,0.41)	<0.001
**BETWEEN GROUPS COMPARISON**			
Natalizumab vs Non-switchers	0.32	(0.13,0.81)	0.017
Immunosuppressant vs Non-switchers	0.24	(0.09,0.64)	0.004
Immunosuppressant vs Natalizumab	0.76	(0.36,1.63)	0.49

Within group comparison compares the relapse rate before and after the switch for each group. The between group comparison compares treatment effect on immunosuppressant and natalizumab groups with non-switchers or between natalizumab and immunosuppressants.

The percentage of patients who remained relapse-free after the switch was 53.8% for the non-switchers, 74.2% for the natalizumab group and 50% for the immunosuppressants group which had the longest follow-up after the switch (p = 0.06).

The analyses were repeated considering the type of disease (SPMS vs. RRMS). For the RRMS patients the within group reduction in the relapse rate was 60% (95%CI 33, 76%, p<0.001) for natalizumab and 78% (95%CI 50, 91%) for immunosuppressant group, while the reduction in the non-switchers was not significant (14%, 95%CI -80, 67%, p = 0.76). Comparing natalizumab to the non-switchers we observed a reduction in the relapse rate of 54% (95%CI -62, 84%, p = 0.17) and in the comparison between immunosuppressant switchers and non-switchers, a reduction of 75% was observed for immunosuppressant switchers (95%CI 7,93%, p = 0.038). Immunosuppressant switchers had a reduction in the relapse rate compared to natalizumab, although this difference was not statistically significant (46%, 95%CI -58,79%, p = 0.21). For the SPMS patients there was a reduction in the relapse rate of 97% (95% CI 62,99%, p = 0.007) after the switch to natalizumab treatment and of 83% after the switch to immunosuppressant treatment (95%CI 35,95%, p = 0.01). Relapse rate reduction in the non-switchers was 6% (p = 0.96). When comparing natalizumab to the non-switchers, there was a reduction of 97% (p = 0.024) and comparing the immunosuppressants to the non-switchers, the reduction was 82% (p = 0.11).

There was a higher proportion of patients treated with IFN-β (61.5% for the non-switchers group, 70% for the natalizumab and 90.9% in the immunosuppressant switchers) than other first-line treatment. We studied the effect of switching in patients who switched from any IFN-β and in those switching from GA. In the IFN-β group, switching to natalizumab was associated with a reduction in the relapse rate of 75% (95%CI 49,87%, p<0.001) and switching to immunosuppressants reduced the relapse rate by 80% (95%CI 61,90%, p<0.001). In this case, the non-switchers had an increase in the relapse rate of 4%, although this difference was not statistically significant (p = 0.93). Comparing the natalizumab group to the non-switchers, natalizumab reduced the relapse rate by 76% (95%CI 21, 92%, p = 0.018). Immunosuppressants reduced the relapse rate by 80% (95%CI 42, 93%, p = 0.003) when comparing to the non-switchers. There were no differences between immunosuppressant and natalizumab switchers (IRR 0.8, 95%CI 0.31, 2.02, p = 0.63).

There were only 25 patients who switched from GA to a second-line MS agents. In those patients previously treated with GA, the switch to natalizumab reduced the relapse rate by 63% (95% CI 15, 84%, p = 0.019) while the switch to immunosuppressant was associated with an increase in the relapse rate by 8% (IRR 1.08, 95% 0.17,6.88, p = 0.94). As this group was small, 95% CI are large.

## Discussion

In this study of 95 patients with breakthrough relapsing MS on first-line DMTs, we found that switching to either natalizumab or immunosuppressant achieved large net reductions in the relapse rate. The reduction in the relapse rate among the natalizumab switchers was similar in magnitude to the effect size reported in the pivotal trial of natalizumab monotherapy, in which the annualized relapse rate was reduced by 68% compared to placebo [2]. Reported rates of relapse reduction in both the mitoxantrone and cyclophosphamide trials were 63% compared to the control groups in the first year [9], [10]. However, these studies included greater proportion patients with SPMS or who received combination therapy.

Although immunosuppressants and natalizumab had a similar impact on reducing the relapse rate in our study, the exposure to strong immunosuppressants may be associated with an increased risk of serious infection or secondary neoplasm [11], [12] or, in the case of mitoxantrone, with cardiotoxicity [9], [13]. While there are risks associated with natalizumab, particularly that of progressive multifocal leukoencephalopathy, such adverse events appear to be fairly infrequent [14], [15]. Some clinicians have considered the use of drug holidays to reduce the risk of developing PML. However this interruption might lead to a return of clinical flares and radiologic activity [16] or at least to the pre-treatment level of disease activity [17]. Active monitoring of patients receiving natalizumab by regular MRI scanning, careful assessment of new MRI lesions or symptoms and, when necessary, CSF analysis is crucial for detecting JC virus infection. Treatment with plasma exchange (PLEX) followed by steroids when there is neurologic progression due to an immune reconstitution inflammatory syndrome (IRIS) is considered the treatment of choice after the diagnosis of PML, although there are no clinical trials [18].

Another question that remains unclear is whether combination therapy is helpful and safe for patients with breakthrough disease on the first-line DMT. Some studies have shown that the combination of first-line and second-line therapies is effective in reducing disease activity [19]. Although using multiple therapies with different mechanisms of action in other diseases supports a similar approach in MS, the evidence for doing so is limited [20]. More studies are needed to clarify the usefulness and safety of combination therapy.

The balance in the risk/benefit ratio of natalizumab versus immunosuppression as second-line therapy is unknown and may be influenced not only by potential adverse effects but also by factors such as drug- and infusion-related costs and ease of access.

While our study addresses an important clinical question, it has some limitations. Since the treatment assignment was not randomized, there might be differences in the groups that led to a different response to second-line therapies. Although the model was adjusted for all known potential confounders, there may be important covariates for which we did not account. In addition, since patients had breakthrough disease before the switch, it is important to consider the possible effect of regression to the mean, which may lead to a falsely high reduction in the relapse rate within groups. However, the three groups had similar relapse rates before switching both overall and in the last year on first-line DMT, such that if disease activity was unusually high in the pre-switch period, one would expect regression to the mean to occur in a non-differential manner during the follow-up period. As such, any difference between groups is likely a true effect rather than a result of regression to the mean, which could only explain differences within groups. Information on progression of disability as documented by EDSS after the switch to second-line DMT was not available for all patients.

Immunosuppressants were available before natalizumab and the factors influencing a decision to switch may have changed over time, leading to differences between the immunosuppressant and natalizumab groups for which we cannot account. A further potential problem is that the threshold for recommending a switch may differ among our center's neurologists, in part due to the lack of definition of breakthrough disease. A standard definition of breakthrough disease should be established in order to promptly identify sub-optimal responders to first-line therapies who may benefit from switching to second-line therapies. In our study, breakthrough was defined clinically and/or radiologically by the patient's MS specialist. As recommended in the literature [21], the monitoring strategies to evaluate DMT effectiveness at our center included regular follow-up visits by the same neurologist, and monitoring of relapses, disease progression by EDSS, and new T2-bright and gadolinium-enhancing lesions on brain MRI scans. We also excluded reasons other than breakthrough disease for stopping first-line DMT such as poor compliance or side effects. Besides this, patients treated with IFNB are tested for neutralizing antibodies to IFNB when suspecting breakthrough disease and considering a switch to another therapy [21].

While second-line therapies are more likely to be associated with rare but serious adverse effects, our data provide strong evidence for switching to such treatments when patients experience breakthrough disease on first-line agents. These results do not negate previous observations that patients may benefit from switching from one to another first-line therapy [3], [4], [5]. Defining an algorithm for the timing and indications for treatment switch would be of great clinical utility but requires a consensus definition of breakthrough disease.

## References

[1] Rudick RA, Stuart WH, Calabresi PA, Confavreux C, Galetta SL (2006). Natalizumab plus interferon beta-1a for relapsing multiple sclerosis.. N Engl J Med.

[2] Polman CH, O'Connor PW, Havrdova E, Hutchinson M, Kappos L (2006). A randomized, placebo-controlled trial of natalizumab for relapsing multiple sclerosis.. N Engl J Med.

[3] Gajofatto A, Bacchetti P, Grimes B, High A, Waubant E (2009). Switching first-line disease-modifying therapy after failure: impact on the course of relapsing-remitting multiple sclerosis.. Mult Scler.

[4] Caon C, Din M, Ching W, Tselis A, Lisak R (2006). Clinical course after change of immunomodulating therapy in relapsing-remitting multiple sclerosis.. Eur J Neurol.

[5] Carrá A, Onaha P, Luetic G, Burgos M, Crespo E (2008). Therapeutic outcome 3 years after switching of immunomodulatory therapies in patients with relapsing-remitting multiple sclerosis in Argentina.. Eur J Neurol.

[6] Putzki N, Yaldizli O, Mäurer M, Cursiefen S, Kuckert S (2010). Efficacy of natalizumab in second line therapy of relapsing-remitting multiple sclerosis: results from a multi-center study in German speaking countries.. Eur J Neurol.

[7] McDonald WI, Compston A, Edan G, Goodkin D, Hartung HP (2001). Recommended diagnostic criteria for multiple sclerosis: guidelines from the International Panel on the Diagnosis of Multiple Sclerosis.. Ann Neurol.

[8] Waubant E, Vukusic S, Gignoux L, Dubief FD, Achiti I (2003). Clinical characteristics of responders to interferon therapy for relapsing MS.. Neurology.

[9] Hartung HP, Gonsette R, König N, Kwiecinski H, Guseo A (2002). Mitoxantrone in progressive multiple sclerosis: a placebo-controlled, double-blind, randomized, multicentre trial.. Lancet.

[10] Smith DR, Weinstock-Guttman B, Cohen JA, Wei X, Gutmann C (2005). A randomized blinded trial of combination therapy with cyclophosphamide in patients with active multiple sclerosis on interferon beta.. Mult Scler.

[11] Radis CD, Kahl LE, Baker GL, Wasko MC, Cash JM (1995). Effects of cyclophosphamide on the development malignancy and on long-term survival of patients with rheumatoid arthritis. A 20-year follow up study.. Arthritis Rheum.

[12] Ellis R, Boggild M (2009). Therapy-related acute leukaemia with mitoxantrone: what is the risk and can we minimize it?. Mult Scler.

[13] Ghalie RG, Edan G, Laurent M, Mauch E, Eisenman S (2002). Cardiac adverse effects associated with mitoxantrone (Novantrone) therapy in patients with MS.. Neurology.

[14] Langer-Gould A, Atlas SW, Green AJ, Bollen AW, Pelletier D (2005). Progressive multifocal leukoencephalopathy in a patient treated with natalizumab.. N Engl J Med.

[15] Food and Drug Administration website. Natalizumab information (marketed as Tysabri).. http://www.fda.gov.

[16] West TW, Cree BAC (2010). Natalizumab dosage suspension: are we helping or hurting?. Ann Neurol.

[17] Clifford DB, DeLuca A, Simpson DM, Arendt G, Giovannoni G (2010). Natalizumab-associated progressive multifocal leukoencephalopathy in patients with multiple sclerosis: lessons from 28 cases.. Lancet Neurol.

[18] Kaufman MD, Lee R, Norton HJ (2010). Course of relapsing-remitting multiple sclerosis before, during and after natalizumab.Mult Scler; Dec 6 (Epub ahead of print)..

[19] Radue EW, Stuart WH, Calabresi PA, Confavreux C, Galetta SL (2010). Natalizumab plus interferon beta-1a reduces lesion formation in relapsing multiple sclerosis.. J Neurol Sci.

[20] Conway D, Cohen JA (2010). Combination therapy in multiple sclerosis.. Lancet Neurol.

[21] Rudick RA, Polman CH (2009). Current approaches to the identification and management of breakthrough disease in patients with multiple sclerosis.. Lancet Neurol.

